# A Rare Case of Pneumopericardium in the Setting of Tuberculous Constrictive Pericarditis

**DOI:** 10.1155/2017/4257452

**Published:** 2017-05-28

**Authors:** Lauro L. Abrahan IV, Stephanie Martha O. Obillos, Jaime Alfonso M. Aherrera, Jose Donato A. Magno, Celia Catherine C. Uy-Agbayani, Ulysses King G. Gopez, Jobelle Joyce Anne R. Baldonado

**Affiliations:** ^1^Section of Cardiology, Department of Medicine, University of the Philippines, Philippine General Hospital, Manila, Philippines; ^2^Department of Medicine, University of the Philippines, Philippine General Hospital, Manila, Philippines; ^3^Division of Thoracocardiovascular Surgery, Department of Surgery, University of the Philippines, Philippine General Hospital, Manila, Philippines

## Abstract

A 28-year-old Filipino male was admitted due to high-grade fevers and dyspnea on a background of chronic cough and weight loss. Due to clinical and echocardiographic signs of cardiac tamponade, emergency pericardiocentesis was performed on his first hospital day. Five days after, chest radiographs showed new pockets of radiolucency within the cardiac shadow, indicative of pneumopericardium. On repeat echo, air microbubbles admixed with loculated effusion were visualized in the anterior pericardial space. Constrictive physiology was also supported by a thickened pericardium, septal bounce, exaggerated respiratory variation in AV valve inflow, and IVC plethora. A chest CT scan confirmed the presence of an air-fluid level within the pericardial sac. The patient was started on a quadruple antituberculosis regimen and IV piperacillin-tazobactam to cover for superimposed acute bacterial pericarditis. Pericardiectomy was performed as definitive management, with stripped pericardium measuring 5–7 mm thick and caseous material extracted from the pericardial sac. Histopathology was consistent with tuberculosis. This report highlights pneumopericardium as a rare complication of pericardiocentesis. We focused on the utility of echocardiography for diagnosing and monitoring this condition on a background of tuberculous constrictive pericarditis, ultimately convincing us that pericardiectomy was necessary, instead of the usual conservative measures for pneumopericardium.

## 1. Introduction

Pneumopericardium is defined as the presence of an air-fluid level in the pericardial sac [1]. Although a rare entity, it can be the consequence of several procedures and conditions, some of which are iatrogenic (pericardiocentesis, open-heart surgery, etc.) in nature. We present a unique case of pneumopericardium in the setting of tuberculous constrictive pericarditis.

## 2. Case Report

A 28-year-old male with no known comorbid illnesses was admitted for a one-week history of night fevers reaching up to 39°C and progressive shortness of breath. Review of systems revealed he had a chronic history of intermittent cough with whitish sputum, as well as significant weight loss (10 kg) over the past month. One of the patient's aunts living in their household was previously diagnosed and treated for pulmonary tuberculosis. He denied any vices.

On physical exam, his blood pressure was stable at 90/60 mm Hg, but the patient was tachycardic (122 beats/minute) and tachypneic (25 breaths/minute). He had no palpable cervical or axillary lymphadenopathy, but neck veins were prominently distended. Breath sounds were decreased at the left base with crackles. The precordium was adynamic with no note of any heaves or thrills. Heart sounds were regular in rhythm and sounded distant. There were no appreciated murmurs, friction rubs, or pericardial knocks. Abdomen was flat and soft with no signs of hepatomegaly or ascites. No pedal edema was noted. Pulsus paradoxus was appreciated with a difference of 12 mm Hg between systolic blood pressure measurements during inspiration and expiration.

**Figure 1:**
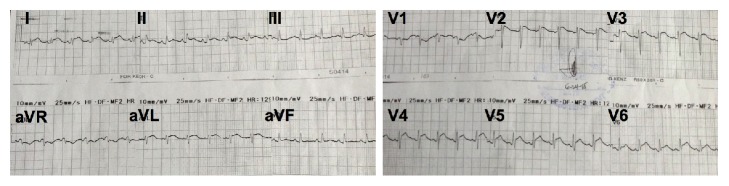
ECG showed diffuse ST elevation and PR segment depression (except in leads aVR and V_1_), consistent with pericarditis.

**Figure 2:**
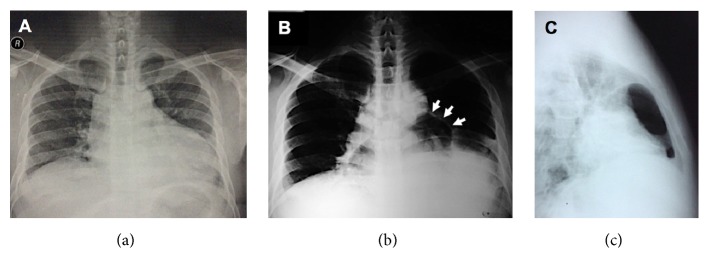
Evolution of the patient's chest radiographs. The prepericardiocentesis film (a) showed cardiomegaly secondary to the massive pericardial effusion. Five days after removal of fluid, new pockets of radiolucency were detected within the cardiac silhouette on both frontal (b) and lateral (c) projections, representing air within the pericardium (arrows pointing at outline of the sac).

The 12-lead ECG demonstrated classic signs of pericarditis (Figure 1) and the chest radiograph revealed cardiomegaly with normal pulmonary vascularity (Figure 2(a)). A focused bedside echocardiogram showed a massive circumferential pericardial effusion with right atrial and right ventricular collapse during diastole. Left ventricular systolic function was preserved with adequate wall motion and an ejection fraction (EF) of 68%. Pulmonary artery pressure was also within normal limits.

The patient was transferred to the ICU where bedside echo-guided subxiphoid pericardiocentesis was performed. Approximately 625 mL of grossly turbid, nonbloody yellow fluid was extracted over the course of 72 hours before the pericardial catheter was removed. Fluid examination yielded low glucose levels (20 mg/dL) with some leukocytes (2473 cells/microliter with neutrophilic predominance) and red blood cells (6680 cells/microliter). The KOH smear was negative for fungal elements, and cytologic analysis did not reveal any malignant cells. The initial Gram stain showed the presence of Gram-positive cocci in pairs and in clusters, which failed to grow on standard culture media, possibly due to previous antibiotic coverage. Adenosine deaminase (ADA) diagnostic kits are not readily available in our country as of this writing, and therefore ADA level analysis was not performed. Sputum acid-fast bacilli (AFB) smears and HIV ELISA screen were negative. After pericardiocentesis, the patient's shortness of breath improved but did not completely resolve. A chest radiograph taken after five days showed new pockets of radiolucency within the cardiac silhouette (Figures 2(b) and 2(c)).

**Figure 3:**
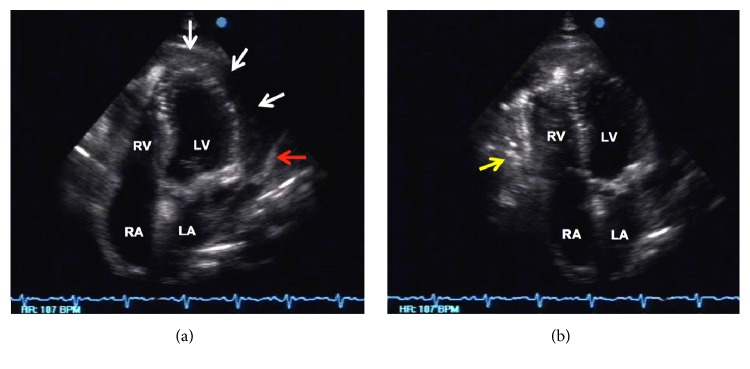
Echocardiographic apical four-chamber views demonstrating pneumopericardium. (a) There was a large (20 mm) circumferential layer (white arrows) within the pericardial space, with its heterogenous nature suggestive of possibly purulent pericardial fluid. Marked thickening of the pericardium (5 mm) can also be appreciated in this view (red arrow). (b) Slight angulation of the probe to focus on the anterior pericardial space (anterior to the right ventricle) revealed the presence of microbubbles (yellow arrow) representing air freely moving within the pericardial sac.

**Figure 4:**
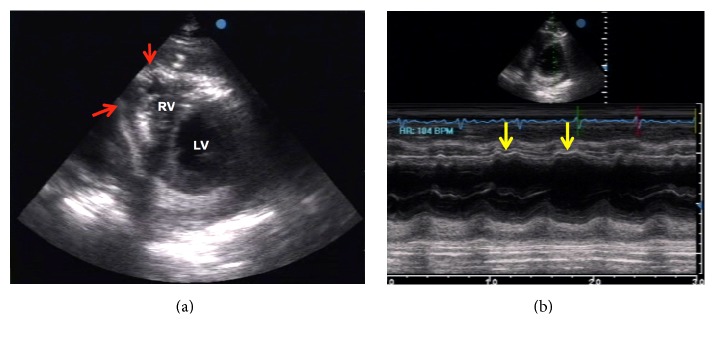
Echocardiographic parasternal short axis views at the level of the mid-left ventricle (LV). (a) The free wall of the right ventricle (RV) appears adherent to the adjacent pericardium (red arrows). There was note of septal bounce, leading to a D-shaped LV cavity during diastole. (b) M-mode of the same view confirmed the wavy motion (yellow arrows) of the interventricular septum representing septal bounce.

**Figure 5:**
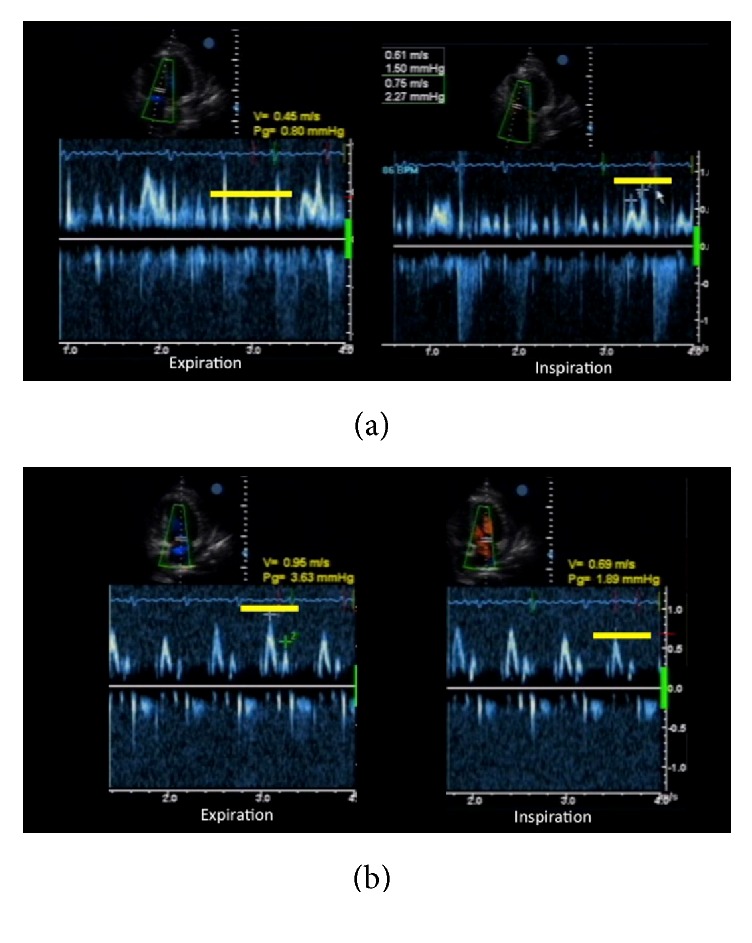
Exaggerated respiratory variations in tricuspid and mitral valve inflow. During inspiration, there was a significant increase in velocity for the tricuspid valve (a), while the mitral valve exhibited decreased inflow velocity (b).

**Figure 6:**
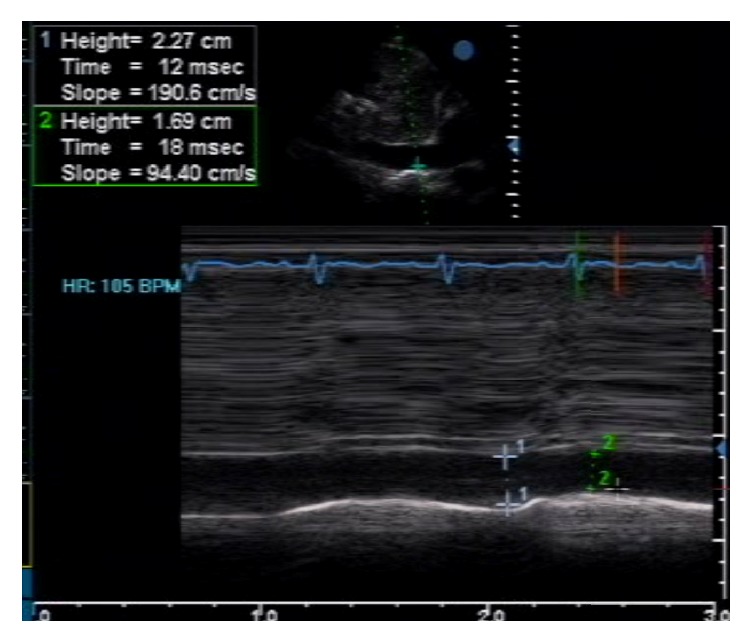
The IVC was dilated (23 mm), with minimal decrease in diameter on inspiration (only 26%), indicative of IVC plethora.

Follow-up echocardiographic studies were done to confirm the presence of pneumopericardium and check for constrictive physiology that would warrant pericardiectomy. Air microbubbles were visualized mainly in the anterior pericardial space (likely due to gravitational effects in the supine position), with a significant amount of reaccumulated pericardial fluid and frond-like structures (Figure 3). Even though diastolic collapse of the right-sided chambers was no longer observed, several indicators of constrictive physiology were demonstrated in this second study, such as marked thickening of the pericardium (Figure 3(a)), septal bounce due to interventricular independence (Figure 4), exaggerated respiratory variation in tricuspid and mitral inflow (Figure 5), and IVC plethora (Figure 6). Although pericardial thickness is better measured on cardiac CT or MRI, the other indicators provided sufficient evidence for pericardial constriction.

**Figure 7:**
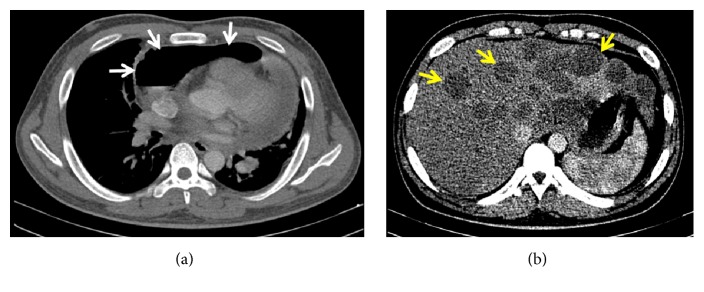
CT scan images. (a) Cross-sectional cut of the heart and pericardium revealed an air-fluid level within the pericardial sac (white arrows). (b) Cross-sectional cut of the liver showed multiple hypodense nonenhancing foci (yellow arrows).

A CT scan with contrast confirmed the diagnosis of pneumopericardium, manifesting as a clearly visible air-fluid level (Figure 7(a)). The study also revealed a left-sided pleural effusion with consolidation and atelectasis of adjacent lung segments, enlarged and confluent para-aortic and pre- and postcarinal lymph nodes, and several hypodense nonenhancing foci throughout the liver (Figure 7(b)). These findings were consistent with a pneumonic process on top of disseminated tuberculous infection involving the pericardium, liver, lymph nodes, and possibly the pleura. Importantly, there were no noted fistulous tracts between the pericardium and trachea/GI tract.

The patient was started on oral quadruple combination anti-TB medications (isoniazid, rifampicin, pyrazinamide, and ethambutol). Intravenous piperacillin-tazobactam was also given for fourteen days to cover for a possible bacterial superinfection on top of the tuberculous pericarditis, as well as possible nosocomial pneumonia. Since the patient already had features of constrictive physiology to begin with, we decided there would be little incremental benefit in administering corticosteroids to prevent progression of constriction, when weighed against the possible risks. Despite almost three weeks of anti-TB medications, the patient still complained of exertional dyspnea. Aware of the possibility of a repeat episode of tamponade from fluid reaccumulation, we decided to proceed with decompressive surgery.

**Figure 8:**
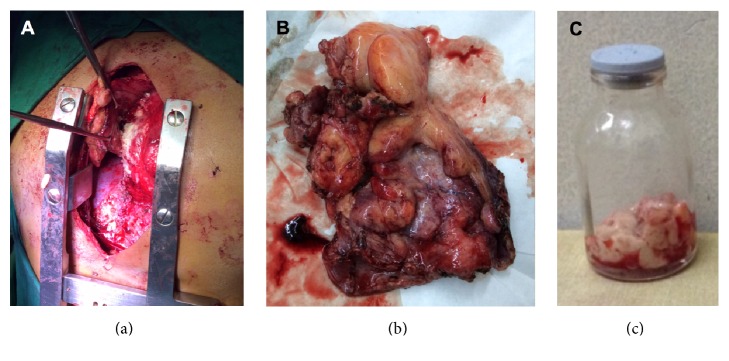
Intraoperative findings. (a) Pericardiectomy through median sternotomy was performed. (b) Stripped pericardium with measured thickness of 5–7 mm. (c) Caseous material extracted from the pericardial sac.

Surgical treatment consisted of pericardiectomy through a median sternotomy (Figure 8(a)). Intraoperatively, the thickness of the stripped pericardium was measured to be 5–7 mm (normal: <2 mm) [2] (Figure 8(b)). Caseous material was extracted from the pericardial sac (Figure 8(c)). Cultures and polymerase chain reaction (PCR) of the pericardial sac contents were negative for* Mycobacterium tuberculosis* and other bacteria. However, histopathology was consistent with acute suppurative pericarditis on top of chronic granulomatous inflammation with Langhans-type giant cells and caseation necrosis, indicative of tuberculous pericarditis with superimposed bacterial pericarditis. AFB culture of the excised pericardial tissue was also positive after two weeks of incubation.

Postoperative echocardiography revealed that constrictive physiology was no longer present, with preserved systolic function in both ventricles. He was discharged improved and completed the prescribed 6 months of anti-TB medications, with resolution of his symptoms and gain in weight back to preillness levels.

## 3. Discussion

### 3.1. Pneumopericardium

The spectrum of etiologies for pneumopericardium is broad, including trauma, complications of procedures, fistulization from adjacent structures, barotrauma, and pericardial infections [3]. The most common cause remains to be trauma after penetrating or blunt chest injury [4].

For our patient, the likely cause was iatrogenic introduction of air during the pericardiocentesis. Fistulization was ruled out through the CT scan. There have been documented reports of pericardial bacterial infections leading to pneumopericardium [5], and initially we could not rule out this possibility, hence the coverage with piperacillin-tazobactam. In retrospect, even with the negative bacterial cultures, this was a prudent decision since the histopathology report was suggestive of a bacterial superinfection.

Table 1 summarizes the salient features of several published case reports on pneumopericardium. Our case shares similar characteristics with some of them, particularly Cases  1 and 7. Our case is unique, however, in that the pneumopericardium occurred on a background of constrictive pericarditis from the patient's disseminated tuberculosis infection. The constrictive features that were demonstrated and lack of improvement with medical treatment steered the patient's management towards definitive pericardiectomy, rather than the conservative measures that are usually implemented in stable patients with normal echocardiographic hemodynamics.

### 3.2. Constrictive Pericarditis

A retrospective study by Roque et al. [11] from the UP-Philippine General Hospital described the profile of 22 admitted patients with constrictive pericarditis over a span of two years. Tuberculosis was identified as the leading etiology in the local setting. On echocardiography, septal bounce and an exaggerated AV inflow pattern during inspiration were the most common findings (64% for both), followed by the presence of concomitant pericardial effusion (54%). The authors recommended pericardiectomy as the mainstay of treatment for this condition.

### 3.3. Echocardiography in Pericardial Diseases

Echocardiography had several vital applications in this case. Firstly, it served as a confirmatory test for the diagnosis of pneumopericardium initially detected on the chest radiograph. While CT scan may be a more accurate test for this purpose, the echocardiogram is a readily available and less costly exam, with diagnostic microbubbles that can easily be detected by an experienced sonographer.

A unique advantage over CT imaging is the capability of echocardiography to monitor for signs of tamponade, a crucial consideration, since pneumopericardium can progress to tamponade [3, 7]. Equally important is the detection of constrictive parameters on echocardiography that would justify removal of the pericardial sac. The lack of tamponade physiology on the second echo exam was our basis for proceeding directly to pericardiectomy to relieve the constriction, instead of having to perform a temporizing pericardiostomy to drain the air/fluid.

Lastly, postoperative echocardiography was able to demonstrate the success of pericardiectomy, with resolution of the constrictive physiology that was seen prior to surgery.

## 4. Conclusion

We have presented a unique case of TB constrictive pericarditis complicated by iatrogenic pneumopericardium. We have demonstrated how the different modalities of echocardiography were used in diagnosis and therapeutic planning for our complex case. Instead of the typical conservative approach to such a complication, the concomitant constriction demonstrated on echo directed us towards definitive pericardiectomy. Knowledge of these complications is of paramount importance to guide the clinician in diagnosis and management.

## Figures and Tables

**Table 1:** Summary of selected case reports on pneumopericardium.

Case	Age/sex	Etiology	Presentation	Management	Outcome
1	20/M [1]	Pericardiocentesis of TB effusion	Pleuritic chest pain	Conservative Steroids TB meds	Improved

2	47/F [6]	Pericardiocentesis in scleroderma	Asymptomatic	Conservative Steroids	Improved

3	69/M [7]	CABG	Cardiac tamponade	Surgical decompression	Improved

4	40/F [8]	Double valve replacement	Cough Pericardial friction rub	Conservative	Improved

5	80/F [3]	Rupture of gastric volvulus into pericardial cavity	STEMI	Palliative	Expired

6	60/F [9]	Barotrauma (mechanical ventilation)	Pneumothorax Pneumomediastinum Desaturation	Pericardiocentesis Needling CPR	Expired

7	54/M [5]	Bacterial infection *(Streptococcus milleri)*	Cardiac tamponade High fever	Pericardiocentesis Antibiotics Intrapericardial urokinase	Improved

8	20/M [10]	Spontaneous	Dyspnea Neck & chest pain Crepitus	Conservative	Improved

TB = tuberculous/tuberculosis; CABG = coronary artery bypass graft; STEMI = ST-elevation myocardial infarction.
